# Condensed Tannins in Drinking Water for Broilers and Their Effects on Intestinal Micrometry, Performance, and Fatty Acid Profile in Meat

**DOI:** 10.3390/vetsci12121125

**Published:** 2025-11-27

**Authors:** Larissa Elen Hirt Bourckhardt, Maiara Sulzbach Marchiori, Bruna Klein, Antony Comin, Charline Marchioro, Jessica Line Farias de Lima, Danielle Dias Brutti, Aleksandro Schafer da Silva

**Affiliations:** 1 Departamento de Zootecnia, Universidade do Estado de Santa Catarina (UDESC), Chapecó 89815-630, Brazil; 2 Programa de Pós-Graduação em Zootecnia, Universidade do Estado de Santa Catarina, Chapecó 89815-630, Brazil; 3 Departamento de Engenharia de Alimentos e Engenharia Química, Universidade do Estado de Santa Catarina, Pinhalzinho 89870-000, Brazil; 4 Centro de Ciências da Agrárias, Universidade Federal do Rio Grande do Sul, Porto Alegre 91540-000, Brazil

**Keywords:** *Acacia mearnsii*, tannins, intestinal health, fatty acids, performance

## Abstract

The study evaluated the effects of including condensed tannins (CT) from *Acacia mearnsii* bark in the drinking water of broilers on performance, intestinal health, and meat quality. A total of 240 male Cobb 500 chickens were distributed into three treatments: negative control (NC, without tannin), NC + 500 g/m^3^ of condensed tannin (CT50), and NC + 700 g/m^3^ of tannin (CT70), with the doses defined based on a pilot study. The experiment showed that CT50 presented better initial performance (d1–21), with greater weight gain and better productive efficiency index (PEI). But when evaluating the initial and growth phases simultaneously (d1–35), we found that birds from CT50 had better feed conversion and PEI compared to NC. When considering the total experimental period (d1–42), there was no effect of the treatment on performance. In the intestine and meat, lower levels of reactive oxygen species and lipid peroxidation were observed in CT50 and CT70 compared to NC, combined with higher levels of non-protein thiols. The CT70 treatment favored meat quality, reducing saturated fatty acids and increasing the concentration of unsaturated fatty acids, ω6, and the UFA/SFA ratio. There were no significant changes in the depth of the intestinal crypts, but the greater height of villi and number of enterocytes in the groups that consumed tannins are indicative of improved intestinal health. The thickness of the intestinal wall was lower in the chickens that consumed the condensed tannin compared to the NC. Lower concentrations of total proteins, albumin, and globulin in the serum were observed in the CT70 when compared to the NC. It is concluded that the use of tannins in the drinking water can be a viable strategy to improve the performance in the initial phase (d1–21) and meat quality of chickens.

## Simple Summary

The role of tannins in chicken feed is well known; however, condensed tannins (CT) through drinking water are rare. Intake of CT via drinking water increased weight gain in chickens. In meat, CT intake reduced oxidation reactions and altered the fatty acid profile, reducing saturated fatty acids and increasing unsaturated fatty acids.

## 1. Introduction

Chicken meat consumption has grown considerably in recent years [1]. This increase reinforces the need to adopt alternatives that can increase poultry productivity and ensure the quality of the final product, which meets the growing demand of the consumer market. In this context, the inclusion of plant extracts in animal nutrition has been widely studied, due to the presence of bioactive compounds that positively influence the metabolism of animals [2]. Among these compounds, tannins have gained prominence for their antioxidant and immunomodulatory properties.

The bark of *Acacia mearnsii* has attracted interest due to its high content of condensed tannins, between 20 and 40%, and proanthocyanidins [3,4]. These compounds have strong antioxidant activity and contribute to intestinal health and metabolic efficiency [5]. Condensed tannins influence the metabolism of chickens by interacting with proteins and digestive enzymes, promoting greater efficiency in the digestion of nutrients. In addition, their antioxidant action protects intestinal and muscle epithelial cells, which can improve meat quality and reduce oxidative stress, resulting in better productive performance [6,7].

Studies have shown that the inclusion of tannins in moderate doses (75 to 100 mg/kg) can improve the health status, productive performance, and meat quality of broiler chickens [8,9]. Furthermore, the inclusion of tannins, especially via drinking water, is a little-studied but promising approach as an alternative to conventional growth promoters. This work, therefore, aimed to evaluate whether the addition of condensed tannins from *A. mearnsii* via drinking water has positive effects on the performance, intestinal health, and meat quality of broiler chickens.

## 2. Materials and Methods

### 2.1. Product

The tested product was purchased from the commercial company SETA, located in the city of Estância Velha-RS. NutreSet^®^ (SETA, Estância Velha-RS, Brazil) is a natural zootechnical additive based on condensed tannins (CT), indicated for use in products intended for animal feed. The extract was obtained from the bark of *Acacia mearnsii*—black wattle through an industrial diffusion process, with an availability specification of at least 735 g CT/kg (73.5% purity).

### 2.2. Animals, Accommodation, and Feeding

The experiment was conducted at the experimental farm (FECEO-UDESC), located in the municipality of Guatambu. The 240 male Cobb 500 chicks were purchased from a commercial hatchery in Chapecó-SC/Brazil, at one day of age. The chicks were housed in the experimental shed immediately after arrival and weighing. They were randomly distributed in a completely randomized design and placed in metabolic cages made of aluminum (50 × 70 cm), each cage being considered an experimental unit. The diet provided was the same for all birds, formulated based on corn and soybeans, according to the recommendations of the Brazilian Poultry and Swine Table [10] (Table 1). Three different formulations were used for the initial (1 to 21 days), growth (22 to 35 days), and finishing (36–42 days) phases. What differed between the treatments was the doses of black acacia extract supplied via water (nipple), which consisted of three inclusion levels: 0 g/m^3^, 500 g/m^3^, and 700 g/m^3^. A calculation was made according to the capacity of the facility’s water tanks, which was 100 L, thus including: 0 g, 50 g, and 70 g of the product, identified as CT50, CT70, and negative control (NC), respectively. The doses were determined after a pilot study. The water in the tanks was homogenized twice a day. The water tanks were cleaned weekly, and the product was diluted again. Since the stability of tannins in water depends on pH, temperature, light, and the presence of oxygen, in order to achieve moderate stability, we maintained the water pH at 6.3 ± 0.06 and the temperature at 12.1 ± 0.32 °C in polyethylene tanks sealed to prevent light and oxygen from entering.

The facility had three floors of cages on each side, totaling 48 cages. Therefore, each treatment had 16 replicates per treatment, with 5 birds per cage. The birds were heated using air conditioners and electric heaters at floor height, under controlled conditions according to the age and the lineage manual. Light management followed the recommendations of the lineage manual. The experiment was carried out over 42 days.

### 2.3. Performance

On days 1, 21, 35, and 42, the birds were weighed using a digital scale, as well as the amount of feed consumed per period (d1–21; d22–35; and d36–42). Using the data obtained, weight gain, feed intake, and feed conversion (feed intake/weight gain) were calculated. The productive efficiency index (PEI) was calculated based on the following equation: PEI = {[(viability, %) × (average weight, kg)]/[(bird age, in days) × (feed conversion)]} × 100; where “viability” is the percentage of live birds in each replicate; “average weight” refers to the average weight of the birds in each replicate; “age” is presented as the number of days in the experimental period analyzed; and “feed conversion” for each pen is the information obtained from the calculation of daily feed consumption/daily weight gain.

### 2.4. Sample Collection

#### 2.4.1. Blood

Blood was collected on day 42 of the experiment using insulin syringes, and samples were collected via the ulnar vein from 16 birds in each treatment. The collected samples were used for biochemical analyses and were placed in tubes. Then, the samples were centrifuged at 3500 rpm for 10 min to obtain serum. The serum obtained was frozen at −20 °C until its subsequent analysis for biochemical analysis.

#### 2.4.2. Intestine

On days 21 and 42 of the experiment, after weighing, one bird per cage was slaughtered for the collection of intestine (jejunum) samples. The birds were euthanized by electrocution, followed by cervical dislocation, in order to comply with the recommendations of the ethics committee on the use of animals. One sample (10 cm) was used for histopathological analyses (micrometry) and stored in containers with 10% formalin, and the other fragment was frozen for analysis of oxidative status.

#### 2.4.3. Muscle

Pectoral muscle samples were collected only on day 42 of the experiment for analysis of total lipids, fatty acid profile, and oxidative status. Muscle samples were collected in a standardized way from the left side and stored in sterile plastic bags. From the time of collection, the samples were kept in a thermal box, and upon arrival at the laboratory, they were stored in a freezer (−20 °C) until laboratory analysis.

### 2.5. Intestinal Morphometry

The intestinal fragments were kept in a 10% buffered formalin solution during the 48 h fixation period. After fixation, the pieces were dehydrated in sequences of ethyl alcohol in increasing concentrations (70, 80, 90, and absolute), followed by diaphanization with xylene and inclusion in histological paraffin blocks. The blocks were cut into micrometers, obtaining six semi-serial, 5 um-thick cross-sections. The slides were stained with HE (hematoxylin and eosin), and finally, the samples were mounted between a slide and a coverslip with Entellan(r) resin (Merk, Darmstadt, Germany). Histological analysis was performed under optical microscopy, using a Nikon Eclipse E200 microscope and using the Image J 1.44p program (Wayne Rasband, National Institutes of Health, Bethesda, MD, USA). Intestinal wall thickness data were collected, and then morphometry of the villi and crypts of 5 portions of the intestine was performed for each serial section, totaling 20 measurements per sample. The villus height measurements were taken from the upper base of the crypt to the apex of the villus, and the crypt diameters were calculated from the basement membrane of each crypt end. Enterocyte height was also measured in 20 cells per sample.

### 2.6. Biochemical Analysis

Biomarkers of energy and protein metabolism were measured using the semi-automatic Bio Plus equipment (Bio-2000^®^, Bioplus Produtos para Laboratórios Ltd., Barueri–SP, Brazil). With this analysis, serum levels of albumin, total protein, cholesterol, and uric acid were evaluated. Commercial kits (Gold Analisa Diagnóstica^®^, Belo Horizonte–MG, Brazil) were used for this purpose. Globulin levels were calculated (total protein—albumin).

### 2.7. Meat Analysis

#### 2.7.1. Lipid Extraction and Fatty Acid (FA) Profile

The extraction was carried out by the Bligh and Dyer [11] method with some modifications, and 1.5 g of chicken meat and feed samples, 0.5 mL of water, 5 mL of methanol, and 2.5 mL of chloroform were added into a 15 mL polypropylene tube, and mechanical shaking was performed for 60 min. Thereafter, 2.5 mL of chloroform and Na_2_SO_4_ 1.5% solution were added to promote a biphasic system. This mixture was shaken for 2 min and then centrifuged for 15 min at 2000 rpm. Lipids obtained from the chloroform phase were submitted to fatty acid analysis.

FA methylation was performed by a transesterification method proposed by Hartman and Lago [12]. To the extracted lipids were added 1 mL of 0.4 M KOH methanolic solution in a test tube and shaken in a vortex for 1 min. Samples were kept in a water bath for 10 min at the boiling point. Subsequently, they were cooled at room temperature, and 3 mL of 1 M H_2_SO_4_ methanolic solution was added, shaken in a vortex, and maintained in a water bath for 10 min. With the samples cooled, it was added to 2 mL of hexane and centrifuged at 2000 rpm for 10 min. Lastly, hexane with the fatty acid methyl esters (FAME) was submitted to a chromatography analysis. For the FAME determination, a gas chromatograph model TRACE 1310 equipped with a flame ionization detector was used (Thermo Scientific, Waltham, MA, USA). One microliter of the sample was injected in a split/splitless injector and operated in 1:10 ratio split mode at 250 °C. Hydrogen was used as carrier gas at a constant flow rate of 1.5 mL/min. FAMEs separation was carried out using an RT 2560 (100 m × 0.25 mm × 0.20 μm of thickness film, Restek, Bellefonte, PA, USA) chromatography column. The initial oven temperature was programmed at 100 °C for 5 min and increased to 180 °C at a rate of 8 °C/min. Then, it was increased to 210 °C at a rate of 4 °C/min, and finally, until 240 °C, it was increased at 20 °C/min and maintained for 20 min in isothermal conditions. The detector temperature was kept constant at 250 °C. The FAME compounds were identified by comparison of experimental retention time with those from an authentic standard (FAME Mix-37, Sigma Aldrich, St. Louis, MO, USA). The results were presented as a percentage of each FA identified in the lipid fraction, considering the chain size equivalent factor of FAME for FID and conversion factor of ester to the respective acid, according to Visentainer and Franco [13].

#### 2.7.2. Oxidative Status

The intestine and meat were washed in SET buffer (0.32 M of sucrose, 1 mM of EGTA, 10 mM of Tris–HCl, and pH 7.4) and homogenized (1:10 *w*/*v*) in the same SET buffer with a Potter–Elvehjem glass homogenizer. The homogenate was centrifuged at 800× *g* for 10 min at 4 °C. Part of this supernatant was used to evaluate parameters of oxidative stress. Protein content in the intestinal and meat homogenate was determined by the method of Coomassie blue G dye, using bovine serum albumin as the standard.

Intestinal and meat ROS levels were determined by the dichlorodihydro-fluorescein (DCFH) oxidation method described by LeBel et al. [14], published in detail by Galli et al. [15], using excitation and emission wavelengths of 485 and 538 nm, respectively, and results were expressed as units per DCF, per mg of protein. As an index of lipid peroxidation, thiobarbituric acid reactive substance (TBARS) formation during an acid-heating reaction was determined in intestine and meat as previously described by Ohkawa et al. [16], adapted by Galli et al. [15]. Malondialdehyde (MDA) solution was used as a reference standard. TBARS levels were determined by the absorbance at 532 nm and were expressed as MDA equivalent (nmol MDA/mg of protein). Intestinal and meat non-protein thiol (NPSH) levels were determined calorimetrically at 412 nm as previously described by Ellman [17] and published in detail by Galli et al. [15]. A cysteine solution was used as a reference standard, and NPSH were expressed as micromoles of SH per gram of tissue.

### 2.8. Statistical Analysis

All data were analyzed using the ‘MIXED procedure’ of SAS (SAS Inst. Inc., Cary, NC, USA; version 9.4), with Satterthwaite approximation to determine degrees of denominator freedom for the fixed effects test. Data were tested for normality and homogeneity of variance using the Shapiro–Wilk and Levene tests, respectively. Since both premises were met, we chose to use a parametric test. To compare groups, we used the Tukey test. Significance was defined when *p* ≤ 0.05.

## 3. Results

The performance results are presented in Table 2. In the initial period (d1–21), there was a greater weight gain of the CT70 birds compared to the NC, combined with the greater PEI of both groups that consumed tannin when compared to the control. The feed conversion (d1–35) demonstrated a lower feed conversion in the CT50 compared to the CN and CT70 groups (*p* = 0.050); in this period, a greater PEI was seen in the CT50 compared to the NC. In the period of 1–42 days, there was no effect of the treatment on performance variables (*p* > 0.05).

Table 3 presents the results of biochemical variables of chickens that consumed tannins via drinking water. Lower levels of total protein due to lower levels of albumin were observed in the serum of chickens in the CT70 group compared to the NC group. Birds that consumed condensed tannins (CT50 and CT70) had lower levels of globulins in the serum compared to the control (*p* = 0.005). There was no effect of treatment on uric acid and cholesterol levels (*p* > 0.05).

Table 4 shows the micrometry of chickens that ingested tannins via drinking water. There was a significant difference between treatments regarding villus height on day 21; we observed that the CT50 group had smaller villi compared to the other treatments (*p* = 0.001). The enterocyte diameter on day 21 and in the CN group was lower than in both treatments (*p* = 0.047). On the other hand, for the same variable on day 42, the tannin groups (CT50 and CT70) had a smaller enterocyte size compared to CN (*p* = 0.050). For intestinal wall thickness, we found lower thickness in birds that consumed tannins on day 21 (NC vs. CT50) and day 42 (NC vs. CT50 and CT70). There was no effect of treatment on crypt depth.

In the intestine and meat, lower levels of reactive oxygen species were observed on days 21 and 42 (CT50 and CT70) compared to NC (Table 5). Lower levels of TBARS were observed in the meat and intestine of CT50 and CT70 chickens on day 42 when compared to the control. In the intestine homogenate of CT50 and CT70 chickens, higher levels of NPSH were observed on days 21 and 42 compared to NC; however, no statistical difference was observed in the meat between groups (Table 5).

Table 6 presents the fatty acid profile of the meat. In general, the CT50 and NC groups presented higher concentrations of total saturated fatty acids in the meat than the CT70 group (*p* = 0.032). The CT50 and CT70 groups presented higher concentrations of total unsaturated fatty acids in the meat when compared to the NC group (*p* = 0.021). The saturated fatty acids that showed significant differences between treatments were C14:0 (*p* = 0.002) and C22:0 (*p* = 0.042), presenting higher concentrations in the CT50 and CT70 groups for both fatty acids. The polyunsaturated fatty acids that showed significant differences between treatments were C18:2n6c with higher concentration in the CT50 and CT70 groups (*p* = 0.050), C20:3n6 with higher concentration in the CT50 and NC groups (*p* = 0.025), and C22:6n3 with higher concentration in the NC group (*p* = 0.013). The other fatty acids did not differ between treatments (*p* > 0.05). There was no significant difference between treatments in the concentrations of total monounsaturated fatty acids and total polyunsaturated fatty acids (*p* > 0.05). There was a significant difference in the UFA/SFA ratio, with the CT50 and CT70 groups showing a higher value (*p* = 0.024). The CT50 and CT70 groups also showed higher concentrations compared to NC in other variables, such as ω6 (*p* = 0.047) and total lipids (*p* = 0.001). Results for the fatty acid profile in the feed were presented in Table S1.

## 4. Discussion

The results of this study highlight the potential of *A. mearnsii* bark tannins as a promising alternative for improving zootechnical performance, intestinal health, and meat quality in broilers, also via water. Tannin-fed subjects showed positive effects on weight gain and feed conversion, especially in the initial phase, corroborating studies that indicate the beneficial effect of a 456 mg/kg dose of these compounds on production performance when the additive was added to feed [18]. This positive effect can be attributed to the ability of tannins to modulate the intestinal microbiota, reducing pathogenic microorganisms and promoting beneficial species, such as *Lactobacillus* spp. and *Bifidobacterium* spp. [19]. Tannins modulate the gut microbiota primarily due to their ability to bind to proteins and lipids in bacterial cell membranes, altering their integrity and hindering the adhesion of pathogenic microorganisms [19]. These compounds can also reduce the proliferation of pathogens by directly affecting their metabolism, in addition to creating a more acidic intestinal environment, which favors the growth of beneficial microorganisms [20]. Additionally, tannins influence the expression of bacterial genes related to antibiotic resistance, promoting a microbial balance that favors digestion and nutrient absorption [21].

The modulation of intestinal morphometry was positive, with greater villus height and enterocyte number in the tannin treatments, indicating an increase in absorptive capacity, which is in line with the findings of Buyse et al. [22], who highlight the relationship between intestinal health and performance. These changes suggest that tannins are recommended for maintaining the integrity of the intestinal mucosa, promoting better digestibility and nutrient absorption [19]. This effect is related to the antioxidant properties of tannins, which help protect intestinal epithelial cells against oxidative stress generated by the intense metabolism of birds [5]. Furthermore, tannins can inhibit the activity of specific bacterial enzymes, such as beta-glucuronidases, which can damage the intestinal barrier. This preservation of the intestinal barrier is crucial for preventing inflammation and improving nutrient absorption, as confirmed by improvements in villi height and enterocyte count observed in groups of birds that ingested tannins via water. Studies have shown that tannin addition can result in improvements in intestinal villi height and enterocyte count, indicating a positive effect on intestinal health and nutrient absorption efficiency, as already highlighted in the literature [23,24,25].

Regarding meat quality, tannin treatments improved lipid composition, with a higher proportion of unsaturated fatty acids (UFA), ω6, and UFA/SFA ratio. These parameters are relevant to human nutrition and health, as they are beneficial for reducing cardiovascular risks and improving meat sensory characteristics [26,27]. The higher proportion of UFA in the meat of the tannin-treated groups can be partly explained by their antioxidant action, which reduces the oxidation of polyunsaturated lipids during poultry metabolism [28]. Furthermore, tannins appear to regulate the expression of genes involved in fatty acid synthesis, favoring the conversion of saturated to unsaturated fatty acids, as observed in other studies [29].

On the other hand, higher doses of tannins may present limitations due to the formation of tannin–protein complexes, which can reduce nutrient availability and, consequently, performance, as reported by Mueller-Harvey [29]. This interaction occurs because tannins have phenolic groups capable of forming electrical and hydrophobic bonds with proteins, hindering protein digestibility [30]. At high doses, this mechanism can cause a temporary deficiency of essential amino acids, impacting bird growth and performance [31]. The greater intestinal wall thickness at higher doses may be a moderate inflammatory response, possibly associated with increased free radicals in the intestinal environment, which overwhelm the body’s natural antioxidant capabilities.

The use of tannins also showed positive effects on the biochemical profile, especially total protein and albumin, reinforcing their role in metabolic efficiency. These results are consistent with studies that associate tannins with improvements in immunity and intestinal barrier integrity [32]. This increase in albumin concentration reflects the improvement in liver function and protein-binding capacity, indicating that tannins not only preserve intestinal integrity but are also detrimental to the liver’s metabolic effort in detoxifying aggressive compounds, such as bacterial toxins [33].

In modern poultry farming, it is already known that tannins, when fed in feed, represent a natural and sustainable alternative to antimicrobial growth promoters (AGPs), especially in systems aimed at reducing antibiotic use. In addition to their antimicrobial and antioxidant properties, tannins offer ecological benefits, such as reducing chemical residues in the environment, when compared to synthetic additives [2,4]. This research demonstrates another route of ingestion, in this case drinking water, for the use of condensed tannins, which has been shown to be beneficial for broilers.

Finally, a more detailed analysis of the mechanisms and comparison with similar studies, such as those by Starčević et al. [8] and Adedokun et al. [34], reinforce the idea that tannins can act multifunctionally. They simultaneously affected gut health, production performance, and meat quality, making them an integrated solution to meet the demands of efficiency and sustainability in poultry farming. However, using water, we had some limitations that need to be considered before the poultry farmer adds condensed tannin to the water: (1) preparing the mixture at intervals of less than seven days; (2) the need to homogenize at least once a day in the tannin reservoir to avoid precipitation or supernatant; and (3) if the tannin separates from the water (forming a slime) while in the drinker piping, it can clog the nipples. Furthermore, we did not measure the water consumption of the birds in each cage, since there was a separate water system for each treatment; this information would complement the results of our research. It is important to make it clear to readers that the drinking water contained tannins throughout the experimental period, but we cannot guarantee that tannin consumption was uniform among the animals due to this last limitation. However, if the water is stirred daily, the tannins remain homogenized, and when consumed by the birds, it plays its role as a performance enhancer only in the initial phase.

## 5. Conclusions

The addition of condensed tannins via drinking water proved to be an effective strategy for improving intestinal health, and consequently, the growth performance in the initial phase (d1–21) and meat quality of broiler chickens. The results indicated that the lowest dose (50 g/100 L) was more effective in the initial and growing phases, resulting in greater weight gain and improved feed conversion, while the higher dose (70 g/100 L) improved meat lipid composition, increasing the concentration of unsaturated fatty acids (ω6) and the UFA/SFA ratio, desirable characteristics from a nutritional and sensory perspective.

## Figures and Tables

**Table 1:** Basal diets used in feeding chickens in the initial phase (1–21 days), growth (22–35 days), and finishing phases (35–42 days).

Composition of Feed (kg/ton)	(d1–21)	(d22–35)	(d35–42)
Corn	551	580	621
Soybean meal	373	337	298
Soy oil	39.0	49.2	49.0
Bicalcium phosphate	12.7	13.0	112
Calcitic limestone	11.4	9.28	8.00
Iodized salt	4.86	4.23	3.95
DL-Methionine—99%	2.91	2.82	2.50
L-lysine—78%	2.03	1.95	2.63
L-threonine—99%	0.50	0.48	0.40
Premix of vitamins and minerals ^1^	2.00	2.00	2.00
**Nutritional Requirements**			
Metabolizable Energy (kcal/kg)	3012	3150	3200
Crude protein (%)	23.79	20.58	18.57
Calcium (%)	0.924	0.758	0.634
Digestible Lysine (%)	1.281	1.124	1.014
Digestible Methionine (%)	0.525	0.461	0.416
Digestible methionine + cysteine (%)	0.948	0.832	0.75
Digestible Threonine (%)	0.846	0.742	0.669
Digestible Tryptophan (%)	0.230	0.202	0.183
Sodium (%)	0.221	0.208	0.197

Note ^1^: Vitamin—A UI/kg > 32,000,000; D3 UI/kg > 8,000,000; E UI/kg > 60,000,000; K3 mg/kg > 6,000,000; B1 mg/kg > 7,200,000; B2 mg/kg > 20,000,000; B6 mg/kg > 9,600,000; B12 µg/kg > 40,000,000; B3 mg/kg > 108,000,000; B5 mg/kg > 36,000,000; and B7 µg/kg > 160,000,000. Mineral—Mn PPM > 140,000,000; Zn ppm > 120,000,000; Fe ppm > 100,000,000; Cu ppm > 16,000,000; I ppm > 1,600,000; If ppm > 600,000.

**Table 2:** Growth performance of chickens that ingested tannins via drinking water.

Groups	Feed Intake, kg	Weight Gain, kg	Feed Conversion	PEI, % ^2^
Day 1–21				
Condensed tannin 50 g/100 L of water (CT50)	1.059	0.801 ^ab^	1.336	299.3 ^a^
Condensed tannin 70 g/100 L of water (CT70)	1.110	0.844 ^a^	1.314	307.3 ^a^
Negative control (NC)	1.060	0.753 ^b^	1.408	247.9 ^b^
SEM	0.064	0.040	0.030	5.750
Value *p* ^1^	0.853	0.050	0.080	0.010
Day 1–35				
Condensed tannin 50 g/100 L of water (CT50)	3.246	2.298	1.41 ^b^	475.5 ^a^
Condensed tannin 70 g/100 L of water (CT70)	3.330	2.245	1.48 ^a^	442.9 ^ab^
Negative control (NC)	3.390	2.302	1.47 ^a^	429.5 ^b^
SEM	0.086	0.077	0.019	4.690
Value *p* ^1^	0.062	0.354	0.050	0.010
Day 1–42				
Condensed tannin 50 g/100 L of water (CT50)	4.300	2.843	1.515	456.3
Condensed tannin 70 g/100 L of water (CT70)	4.400	2.828	1.564	442.5
Negative control (NC)	4.402	2.894	1.526	463.9
SEM	0.097	0.049	0.023	5.970
Value *p* ^1^	0.568	0.823	0.198	0.282

Note ^1^. Demonstrates the effect of the treatment, where different letters (^a,b^) in the same column show a significant difference (*p* ≤ 0.05) between the groups. Note ^2^. PEI—Production efficiency index = {[(viability, %) × (average weight, kg)]/[(bird age, in days) × (feed conversion)]} × 100.

**Table 3:** Biochemistry of chickens subjected to tannins via drinking water.

Groups	Albumin (g/dL)	Uric Acid (mg/dL)	Total Protein (g/dL)	Cholesterol (mg/dL)	Globulin (g/dL)
Condensed tannin 50 g/100 L of water (CT50)	1.288 ^ab^	4.325	3.238 ^ab^	118.2	1.950 ^b^
Condensed tannin 70 g/100 L of water (CT70)	1.125 ^b^	3.513	2.950 ^b^	128.5	1.825 ^b^
Negative control (NC)	1.513 ^a^	4.313	3.925 ^a^	124.0	2.413 ^a^
SEM	0.098	0.205	0.120	4.596	0.103
Value *p* ^1^	0.050	0.1470	0.001	0.2583	0.005

NOTE ^1^. Demonstrates the effect of the treatment, where different letters in the same column show a significant difference (*p* ≤ 0.05) between the groups.

**Table 4:** Intestinal micrometry (jejunum) of chickens that ingested tannins via drinking water.

Variables	Days	CT50 ^2^	CT70 ^2^	NC ^2^	SEM	*p*-Value ^1^
Crypta (µm)	21	50.58	49.64	52.88	1.013	0.847
	42	49.02	50.32	54.52	1.080	0.214
Villo (µm)	21	619.6 ^b^	970.8 ^a^	958.0 ^a^	6.045	0.001
	42	1072	1026	1015	5.978	0.968
Enterocyte (number)	21	28.25 ^a^	27.03 ^a^	24.23 ^b^	0.437	0.047
	42	23.62 ^b^	25.28 ^ab^	26.68 ^a^	0.431	0.050
Intestinal wall (µm)	21	839.2 ^b^	1375 ^a^	1443 ^a^	8.198	0.001
	42	1449 ^b^	1458 ^b^	2315 ^a^	8.121	0.001

NOTE ^1^. Demonstrates the effect of the treatment, where different letters on the same line show a significant difference (*p* ≤ 0.05) between the groups. NOTE ^2^. The groups were identified as follows: Condensed tannin 50 g/100 L of water (CT50), condensed tannin 70 g/100 L of water (CT70), and negative control (NC).

**Table 5:** Oxidative biomarkers in the intestine and meat of chickens that ingested tannins via drinking water.

Variables	Day	CT50 ^2^	CT70 ^2^	NC ^2^	SEM	*p*-Value ^1^
Intestine						
ROS (U DCF/mg/protein)	21	241 ^b^	234 ^b^	275 ^a^	6.41	0.01
	42	263 ^b^	258 ^b^	321 ^a^	7.95	0.01
TBARS (nmol MDA/mg of tissue)	21	21.6	23.4	25.1	2.32	0.25
	42	26.1 ^b^	24.4 ^b^	33.7 ^a^	1.98	0.01
NPSH (μmol SH/g of tissue)	21	0.98 ^a^	1.09 ^a^	0.75 ^b^	0.03	0.01
	42	0.86 ^a^	0.89 ^a^	0.72 ^b^	0.03	0.05
**Meat**						
ROS (U DCF/mg/protein)	42	78.6 ^b^	65.7 ^c^	89.5 ^a^	2.05	0.01
TBARS (nmol MDA/mg of tissue)	42	12.8 ^b^	11.3 ^b^	19.7 ^a^	1.23	0.01
NPSH (μmol SH/g of tissue)	42	0.46	0.51	0.43	0.04	0.08

NOTE ^1^. Demonstrates the effect of the treatment, where different letters on the same line show a significant difference (*p* ≤ 0.05) between the groups. NOTE ^2^. The groups were identified as follows: Condensed tannin 50 g/100 L of water (CT50), condensed tannin 70 g/100 L of water (CT70), and negative control (NC).

**Table 6:** Fatty acid profile in meat from chickens that consumed tannins via drinking water.

Fatty Acid (%)	CT50 ^2^	CT70 ^2^	NC ^2^	SEM	*p*-Value ^1^
C12:0 (Lauric)	0.02	0.02	0.03	0.002	0.214
C14:0 (Myristic)	0.28 ^a^	0.25 ^b^	0.29 ^a^	0.004	0.002
C14:1 (Myristoleic)	0.03	0.02	0.03	0.002	0.541
C15:0 (Pentadecanoic)	0.08	0.08	0.08	0.001	0.974
C16:0 (Palmitic)	22.6	22.5	23.0	0.108	0.471
C16:1 (Palmitoleic)	1.19	1.10	1.11	0.038	0.247
C17:0 (Heptadecanoic)	0.22	0.21	0.22	0.002	0.854
C18:0 (Stearic)	12.97	12.60	13.05	0.118	0.456
C18:1n9c (Oleic)	26.0	25.8	25.7	0.157	0.914
C18:2n6c (Linoleic)	27.0 ^ab^	28.0 ^a^	26.4 ^b^	0.125	0.050
C20:0 (Arachidic)	0.18	0.17	0.18	0.002	0.894
C18:3n6 (Linolenic)	0.20	0.19	0.19	0.002	0.935
C20:1n9 (cis-11-Eicosenoic)	0.25	0.23	0.24	0.002	0.076
C18:3n3 (a-Linolenic)	1.32	1.42	1.27	0.022	0.084
C20:2 (cis-11,14-Eicosadienoic)	0.75	0.73	0.76	0.008	0.120
C22:0 (Behenic)	0.15 ^a^	0.13 ^b^	0.15 ^a^	0.003	0.042
C20:3n6 (cis-8,11,14-Eicosatrienoic)	0.61 ^ab^	0.60 ^b^	0.64 ^a^	0.009	0.025
C22:1n9 (Erucic)	0.06	0.03	0.05	0.003	0.203
C20:4n6 (Arachidonic)	5.12	4.83	5.46	0.090	0.359
C22:2 (cis-13,16-Docosadienoic)	0.04	0.03	0.04	0.001	0.247
C24:0 (Lignoceric)	0.10	0.09	0.10	0.002	0.893
C20:5n3 (cis-5,8,11,14,17-Eicosapentaenoic)	0.11	0.09	0.09	0.002	0.148
C24:1n9 (Nervonic)	0.11	0.10	0.12	0.003	0.284
C22:6n3 (cis-4,7,10,13,16,19-Docosahexaenoic)	0.57 ^b^	0.61 ^b^	0.73 ^a^	0.017	0.013
**Variables other**					
∑ Saturated fatty acids (SFA)	36.59 ^ab^	36.10 ^b^	37.15 ^a^	0.109	0.032
∑ Unsaturated fatty acids (UFA)	63.41 ^ab^	63.90 ^a^	62.85 ^b^	0.109	0.021
∑ Monounsaturated fatty acids (MUFA)	27.69	27.38	27.31	0.190	0.924
∑ Polyunsaturated fatty acids (PUFA)	35.72	36.52	35.55	0.206	0.127
UFA/SFA	1.73 ^ab^	1.77 ^a^	1.69 ^b^	0.013	0.024
∑ ω6	32.94 ^ab^	33.64 ^a^	32.66 ^b^	0.108	0.047
∑ ω3	2.00	2.12	2.09	0.023	0.204
ω6/ω3	16.53	15.89	15.65	0.142	0.216
Total lipids	1.17 ^ab^	1.22 ^a^	1.09 ^b^	0.027	0.001

NOTE ^1^. Demonstrates the effect of the treatment, where different letters on the same line show a significant difference (*p* ≤ 0.05) between the groups. NOTE ^2^. The groups were identified as follows: Condensed tannin 50 g/100 L of water (CT50), condensed tannin 70 g/100 L of water (CT70), and negative control (NC).

## Data Availability

The data are with the authors and may be made available upon request with justification; since this is original research and our research group continues to explore the topic in new experiments.

## Supplementary Materials

The following supporting information can be downloaded at https://www.mdpi.com/article/10.3390/vetsci12121125/s1; Table S1: Fatty acid profile in poultry feed during the experimental period.
