# Supplementary material for: Condensed Tannins in Drinking Water for Broilers and Their Effects on Intestinal Micrometry, Performance, and Fatty Acid Profile in Meat
Source: Vet Sci. 2025 Nov 27;12(12):1125. doi: 10.3390/vetsci12121125 (PMC12737662; doi:10.3390/vetsci12121125)
Supplement: Supplementary file 1 [file vetsci-12-01125-s001.zip › vetsci-3953637-supplementary.pdf]

Table S1: fatty acid profile in poultry feed during the experimental period.

**Fatty acid**	**%**
C14:0 (Myristic)	0.101
C15:0 (Pentadecanoic)	0.026
C16:0 (Palmitic)	14.46
C16:1 (Palmitoleic)	0.119
C17:0 (Heptadecanoic)	0.116
C17:1 (cis-10-Heptadecenoic)	0.044
C18:0 (Stearic)	4.846
C18:1n9c (Oleic)	27.00
C18:2n6c (Linoleic)	47.91
C20:0 (Arachidic)	0.474
C18:3n6 (?-Linolenic)	0.094
C20:1n9 (cis-11-Eicosenoic)	0.230
C18:3n3 (a-Linolenic)	3.741
C20:2 (cis-11,14-Eicosadienoic)	0.043
C22:0 (Behenic)	0.489
C22:1n9 (Erucic)	0.013
C22:2 (cis-13,16-Docosadienoic)	0.021
C24:0 (Lignoceric)	0.259
**Other variables**	
∑ Saturated fatty acids (SFA)	20.77
∑ Unsaturated fatty acids (UFA)	79.22
∑ Monounsaturated fatty acids (MUFA)	27.41
∑ Polyunsaturated fatty acids (PUFA)	51.80
UFA/SFA	3.814
∑ ω6	48.02
∑ ω3	3.741
ω6/ω3	12.85
